# Metoprolol and Its Degradation and Transformation Products Using AOPs—Assessment of Aquatic Ecotoxicity Using QSAR

**DOI:** 10.3390/molecules26113102

**Published:** 2021-05-22

**Authors:** Melanie Voigt, Indra Bartels, Dorothee Schmiemann, Lars Votel, Kerstin Hoffmann-Jacobsen, Martin Jaeger

**Affiliations:** 1 Department of Chemistry and ILOC, Niederrhein University of Applied Sciences, Adlerstraße 32, D-47798 Krefeld, Germany; Melanie.voigt@hs-niederrhein.de (M.V.); indra.bartels@stud.uni-due.de (I.B.); Dorothee.schmiemann@hs-niederrhein.de (D.S.); lars.votel@hotmail.de (L.V.); kerstin.hoffmann-jacobsen@hs-niederrhein.de (K.H.-J.); 2 Faculty of Chemistry, University Duisburg-Essen, Universitätsstraße 2, D-45141 Essen, Germany

**Keywords:** ecotoxicity, AOPs, HPLC-HRMS, Metoprolol, QSAR

## Abstract

Pharmaceuticals are found in waterbodies worldwide. Conventional sewage treatment plants are often not able to eliminate these micropollutants. Hence, Advanced Oxidation Processes (AOPs) have been heavily investigated. Here, metoprolol is exposed to UV irradiation, hydrogen peroxide, and ozonation. Degradation was analyzed using chemical kinetics both for initial and secondary products. Photo-induced irradiation enhanced by hydrogen peroxide addition accelerated degradation more than ozonation, leading to complete elimination. Degradation and transformation products were identified by high-performance liquid-chromatography coupled to high-resolution higher-order mass spectrometry. The proposed structures allowed to apply Quantitative Structure-Activity Relationship (QSAR) analysis to predict ecotoxicity. Degradation products were generally associated with a lower ecotoxicological hazard to the aquatic environment according to OECD QSAR toolbox and VEGA. Comparison of potential structural isomers suggested forecasts may become more reliable with larger databases in the future.

## 1. Introduction

Metoprolol, a beta-blocker and one of the most commonly prescribed blood pressure medicines, is introduced into the water cycle through incomplete elimination in sewage treatment plants and is therefore most frequently detected in surface waters worldwide [1,2]. Deleterious effects on fish, invertebrates, and green algae were reported [3,4]. The European Union Directive 93/67EEC classified metoprolol as harmful (10 < EC_50_ < 100 mg L^−1^) to aquatic organisms. The database “Pharmaceuticals in the Environment” of the German Federal Environment Agency records over 1750 entries about the presence in different water bodies of metoprolol worldwide in the year 2018. In Asia, the highest metoprolol concentrations in surface water were detected, amounting to several micrograms per liter [1,2,5]. Similar concentrations were also found in wastewater treatment plant (WWTP) effluents in Western Europe. Maszkowska et al. reported on metoprolol’s stability to hydrolysis, bioavailability, and mobility in the environment [6]. In their studies, they found minor hazardous effects on several marine and soil bacteria, green algae, and duckweed but stated that many more data were needed with respect to long-term, chronic, and synergistic effects. [4]. To minimize environmental hazards, various technologies are being explored worldwide for the implementation of an advanced purification extension to WWTPs, such as the use of activated carbon, bioreactors, membrane reactors, or constructed wetlands [7,8]. Another approach is the use of Advanced Oxidation Processes (AOPs) [9,10,11]. These comprise ozonation, UV radiation treatment, and the use of photocatalysts such as Photo-Fenton, hydrogen peroxide or titanium dioxide. A recent comprehensive survey on the UV based processes and their technical upscaling was conducted by Collivignarelli et al. [12]. The AOPs lead to the formation of hydroxyl radicals that are able to destroy organic micropollutants, but the hydroxyl radicals can also attach themselves to the substances, leading to even more hazardous products [13,14].

Unfortunately, reference standards are very rarely or not at all available for the characterization of the degradation products resulting from these processes. Their synthesis is often costly and complex. As a consequence, only inadequate toxicity data are retrievable for such degradation products. This is also the case for metoprolol. A cost-effective option is quantitative structure-activity relationship (QSAR) analysis [15,16]. QSAR relates the molecular structure with its pharmacological, physico-chemical, toxicological, or ecotoxicological effects. For this purpose, a correlation or a model is created from various data that resulted from in vitro or in vivo ecotoxicologically relevant assays on organisms specific for the environmental compartment. Based on the model established from chemometric or mathematical methods, such as linear regression types, artificial intelligence and machine learning algorithms, ecotoxicity or other sought parameters of not-tested compounds or novel putative molecular structures are predicted.

In this study, solutions of metoprolol were exposed to UV-irradiation at different pH values in the presence and absence of hydrogen peroxide. The resulting degradation and transformation products were identified using high-performance liquid-chromatography coupled with high-resolution mass spectrometry (HPLC-HRMS). Their concentration-time curves were monitored and analyzed by chemical kinetics. In addition, ozonation of metoprolol was performed in saturated ozone solution and under continuous ozone flow. The AOPs and different conditions were compared with respect to the resulting degradation and transformation products. For the UV-irradiation and ozonation products, QSAR analysis was performed using OECD QSAR toolbox (https://qsartoolbox.org/; accessed on 14 May 2021) and VEGA software (https://www.vegahub.eu/; accessed on 14 May 2021) and the ecotoxicity predicted [17,18,19,20]. 

Results from both prediction tools were critically discussed as to the suitability of the AOP for metoprolol elimination from the aquatic environment.

The ecotoxicity of individual degradation or transformation products is often impossible to evaluate experimentally, since standards for the identified products cannot be bought and the synthesis is too expensive. It is also often not known what long-term effects the substances will exercise on the microorganisms. In these cases, QSAR analysis offers a suitable means to predict ecotoxicological potential.

## 2. Materials and Methods

### 2.1. Chemicals and Reagents

Metoprolol tartrate was acquired from Alfa Aesar (98%, Karlsruhe, Germany) and used for all degradation experiments. The eluents for HPLC were A MilliQ water (Millipore System Simplicity 185) and B acetonitrile (99.95%, Carl Roth GmbH + Co. KG, Karlsruhe, Germany), both acidified with formic acid (98–100%, Merck KGaA, Darmstadt, Germany) to 0.1% final concentration. Sample solutions were prepared containing 20 mg L^−1^ metoprolol in MilliQ water. For pH dependent assays, the pH was adjusted with hydrochloric acid (HCl, 30% Suprapur, Merck KGaA, Darmstadt, Germany) or ammoniacal solution (NH_3_, approximately 25% Riedel-de Haen, Seelze, Germany).

### 2.2. Photodegradation Experiments

Photoinduced degradation experiments were carried out in a 1 L batch reactor (Peschl Ultraviolet GmbH, Mainz, Germany). A mercury low pressure VUV/UVC lamp (Heraeus TNN 15/32, 15 W, Hanau, Germany) was located in the middle of the reactor. The UVC lamp emitted polychromatic light whose maximum radiation intensities were found at wavelengths 185, 254, 313, 365, 405, 437, 547, 578, and 580 nm. The total flux of photons in the wavelength range between 200 and 500 nm was determined by means of ferrioxalate actinometry according to Kuhn et al. and Hatchard et al. and amounted to 2.03 mmol·min^−1^·L^−1^ [21,22]. The occurrence of hydroxyl radicals on irradiation of water was proven by Electron Spin Resonance (ESR) spectroscopy using spin traps (data not shown) according to Kochany et al. [23,24]. 

The reactor was filled with 800 mL of the metoprolol solution. Irradiation with UVC light was applied for 10 min. A magnetic stirrer (500 rpm) was used for continuous homogenization in the reactor. The reaction temperature throughout the reactor was 22 ± 2 °C and was checked during degradation by means of a thermometer. Samples were taken every 30 s during five minutes and every minute until ten minutes. 

The photocatalytic degradation of metoprolol was carried out using hydrogen peroxide (30% stabilized, Carl Roth GmbH + Co. KG, Karlsruhe, Germany) that was added to the metoprolol solutions such that concentrations of 10 mg∙L^−1^ and 30 mg∙L^−1^ resulted. Peroxide concentration were determined using Merckoquant test strips (Merck KGaA, Darmstadt, Germany) before and after irradiation.

### 2.3. Ozonation

A 1 L-batch reactor (DWK Life Sciences, Wertheim, Germany) was used for ozonation experiments. Ozone was produced using a COM-AD-01/02 ozone generator (Anseros, Tuebingen, Germany). The oxygen flow was set to 25 L∙h^−1^ and the generator capacity was 2.8%. The ozone flow was continuously led through a glass frit into the reactor containing 1 L of a metoprolol solution with a concentration of 20 mg∙L^−1^. Ozonation proceeded for 30 min while stirring the solution at 500 rpm. Samples were taken every minute. For experiments in ozone-saturated solution, 0.5 L MilliQ water were treated with ozone for 18 min prior to metoprolol addition. Levels of saturation were checked photometrically. A stock solution of metoprolol was added to the solution such that a final metoprolol concentration of 20 mg∙L^−1^ was achieved. Samples were taken every second minute. In all experiments, temperature was kept at 23 ± 2 °C. 

### 2.4. HPLC-HRMS

Reversed-phase chromatographic analysis was performed using a Agilent 1200 Series HPLC system (Agilent Technologies, Inc., Waldbronn, Germany) equipped with an Eclipse Plus C18 (ZORBAX, 3.5 µm, 2.1 × 150 mm, Agilent Technologies, Inc., Waldbronn, Germany). A flow rate of 0.3 mL min^−1^ and a column temperature of 40 °C were used. Elution started with eluents A and B going from 99:1 to 70:30 within 1 min, followed by isocratic conditions of A and B 25:75 during the next 10 min. At 11.1 min, the solvent composition was set to 1:99 and hold for 0.1 min. At minute 15, the gradient was reset to starting conditions and held for further 15 min. For structure identification and recording concentration-time (*c-t*) curves, the HPLC system was coupled to an electrospray ionization (ESI) quadrupole time-of-flight mass spectrometer Agilent 6530 (Q-TOF-MS, Agilent Technologies, Inc., Waldbronn, Germany) with a Dual AJS electrospray ionization interface. The interface capillary temperature was set to 300 °C and the gas flow to 8 L∙min^−1^. The fragmentor voltage was set to 125 V. Spectra were recorded in the positive ion mode with a mass range from 100 to 1000 *m*/*z* at a scan rate of 1 spectrum s^−1^. Both instruments were controlled using MassHunter Workstation B.06.00 (Agilent Technologies, Inc., Waldbronn, Germany).

### 2.5. Kinetic Analysis of the Photodegradation

The degradation curves and kinetic profiles of the degradation products were calculated and fitted mathematically using the curve fitting toolbox within the software MatLab R2020a (MathWorks, Natick, MA, USA). All kinetic profiles and degradation curves were computed according to first-order kinetic models, consecutive and subsequent follow-up reaction models [25,26,27,28,29]. 

### 2.6. Assessment of Ecotoxicology

For QSAR analysis, the software OECD QSAR Toolbox 4.3.1 and the software VEGA version 1.1.5-b36 were used. Calculations in the QSAR Toolbox were based on the Ecological Structure Activity Relationships (ECOSAR) predictive model for aquatic toxicity. For acute ecotoxicity (LC_50_ and EC_50_) and chronic toxicity (ChV) prediction, the organisms *branchiopoda*, *Actinopterygii,* and *green algae* were selected. In addition, a model developed by Veith et al. and Pavan et al. was implemented in the OECD QSAR toolbox [17,18]. From this model, *fathead minnow* and acute toxicity (LC_50_) were chosen. Acute and chronic toxicity were also predicted using VEGA Version 1.1.5-b36. Models from the Instituto di Ricerche Farmacologiche Mario Negri (IRFMN) in Milan, Italy, the United States Environmental Protection Agency (EPA), National Institute of Chemistry (NIC), the project “Development of Environmental Modules for Evaluation of Toxicity of pesticide Residues in Agriculture” (DEMETRA), KNN Read across and ProtoQSAR were included therein. Fish in general, fathead minnow, *Daphnia magna* and algae were selected as representative organisms for the assessment of aquatic toxicity. 

## 3. Results and Discussion

### 3.1. Photoinduced Degradation of Metoprolol—Kinetic Modelling of Degradation

**Figure 1:**
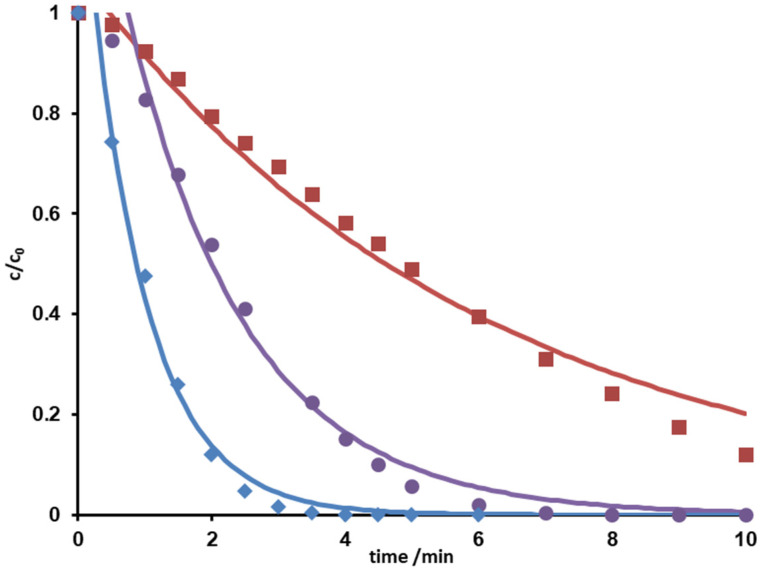
Normalized concentration-time curves of metoprolol (red, ■), in the presence of 10 mg∙L^−1^ H_2_O_2_ (violet, ●) and 30 mg∙L^−1^ H_2_O_2_ (blue, ♦).

The concentration of metoprolol decreased upon UV irradiation. Concentration-time curves (*c-t*) showed a degradation, which could be best described by using first or pseudo-first order chemical kinetics [10,28,29]. The addition of hydrogen peroxide led to accelerated degradation. The corresponding *c-t* curves with increasing concentrations of H_2_O_2_ are given in Figure 1.

It can be clearly seen that increasing hydrogen peroxide concentrations favor the elimination of metoprolol. Reaction rate constants and half-lives indicated an acceleration by a factor of about 6, cf. Table 1. 

The complete degradation of metoprolol was observed after 4.5 min UVC irradiation in the presence of 30 mg∙L^−1^ hydrogen peroxide. This was traced back to the increase of the hydroxyl radical concentration resulting from photolysis of hydrogen peroxide. Increasing the pH from acid to basic caused a slight deceleration of the photo-induced degradation.

### 3.2. Photoinduced Degradation of Metoprolol—Product Characterization

Despite the different photoinduced degradation conditions, similar main intermediate products were observed. Photoinduced degradation follows two major mechanisms: either photolysis by absorption of radiation, excitation and photoreaction, or reaction with hydroxyl radicals formed from UV light induced hydrolysis. A list of all observed and structurally identified intermediates are collected in Table 2.

Products from both mechanisms could be identified. The absorption pathway led to degradation products possessing smaller masses, such as the product with *m*/*z* = 134 and 252. The second mechanism resulted in products with higher masses due to the addition or insertion of hydroxyl radicals, such as *m*/*z* = 284, 282 and 254. These products would be degraded further. Mainly hydroxyl radical induced degradation products were observed. Products with *m*/*z* = 284 and *m*/*z* = 282 were found at different retention times. This is indicative for isomers, as the addition or insertion may take place at different positions. Further investigations using MS^n^ experiments are required for elucidation of the isomers as was described earlier [32,34,35,38,40,44,45,46]. The addition and insertion of hydroxyl radicals seemed to occur predominantly at the aromatic ring and the adjacent carbon atoms. This is in agreement with preliminary results of quantum-chemical calculations showing that the highest electron density includes that region and the highest occupied molecular orbital (HOMO) and the lowest unoccupied molecular orbital (LUMO) extend over that region. Hence, oxidation should be favored there.

**Figure 2:**
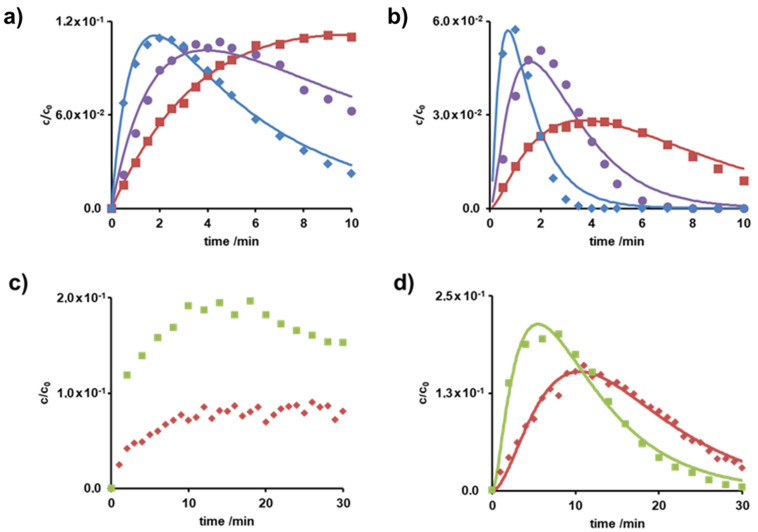
Normalized *c-t*-curves of photoinduced degradation products with (**a**) *m*/*z* = 134 and (**b**) *m*/*z* = 284 RT 4.1 min with 0 mg L^−1^ H_2_O_2_ (red, ■), with 10 mg L^−1^ H_2_O_2_ (violet, ●) and 30 mg L^−1^ H_2_O_2_ (blue, ♦) and normalized *c-t*-curves of degradation or transformation products through ozonation with (**c**) *m*/*z* = 134 and (**d**) *m*/*z* = 300 RT 5.4 min using ozone flow (red, ♦) and in ozone-saturated solution (green, ■).

The *c-t* diagrams of the degradation and transformation products at pH 6 in the presence and absence of hydrogen peroxide are displayed in Figure 2.

The recorded *c-t*-curves were best described by sequence or subsequence follow-up reaction of first-order [25,28]. 

As for the initial compound, hydrogen peroxide induced a faster increase and decrease of the products. Almost all products occurred and vanished within 10 min, except for that with *m*/*z* = 134. The products with *m*/*z* = 134 and 284 are exemplarily shown. In summary, photo-irradiation in combination with hydrogen peroxide proved efficient both for metoprolol itself and for the secondary products. 

### 3.3. Ozonation of Metoprolol

**Figure 3:**
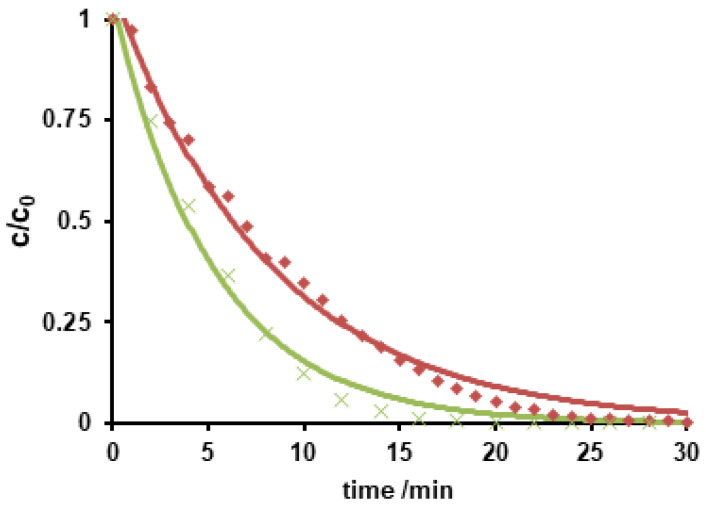
Normalized *c-t*-curves of ozonation of metoprolol using ozone-flow (red, ♦) and in ozone-saturated solution (green, ■).

The *c-t* curves of the degradation of metoprolol upon ozonation are shown in Figure 3.

Monitoring of metoprolol degradation when the compound was added to a previously ozone-saturated solution and when bubbling ozone through a metoprolol containing solution led to different reaction rate constants. As expected, the pre-saturated ozone solution caused a faster degradation. A reaction rate of 0.19 min^−1^ was found while a rate constant of 0.12 min^−1^ was observed in case of ozone bubbling. Both *c-t* curves were fitted using pseudo-first order kinetics since ozone was assumed in excess. 

The same degradation or transformation products were identified as would be expected. An overview of the predominantly formed products is given in Table 3.

As compared to photo-induced degradation, identical products such as *m*/*z* = 282 and *m*/*z* = 134 were found. Yet, different products, such as *m*/*z* = 300, *m*/*z* = 274 and *m*/*z* = 206, were identified as well. Interestingly, structures with the aromatic ring opened were observed, e.g., *m*/*z* = 274 and 206. Products with higher molecular weight than metoprolol were detected more often than after irradiation. 

The concentration of the secondary products was higher when metoprolol was added to an ozone-saturated solution. Thus, formation occurred seemingly faster followed by degradation, since reactions in the ozone-saturated solution were likely limited by mass transfer, whereas degradation in the presence of ozone-flow follow molecular kinetics. In addition, secondary compounds, that were formed, *m*/*z* = 134 (Figure 2c) and 206 (data not shown), but did not react further within 30 min, were observed. In contrast, products with *m*/*z* = 282 (data not shown) and 300 (Figure 2d) were formed and disappeared within 30 min. Comparison of all *c-t* curves suggested that for the elimination of metoprolol and its degradation products, UV-irradiation in combination with hydrogen peroxide might be the more efficient AOP. With respect to installation in WWTP, UV-irradiation based technical processes often suffer from high capital and operative costs and the limited or absent experience of full-scale plant management [12].

### 3.4. Assessment of Ecotoxicity with QSAR Analysis

**Figure 4:**
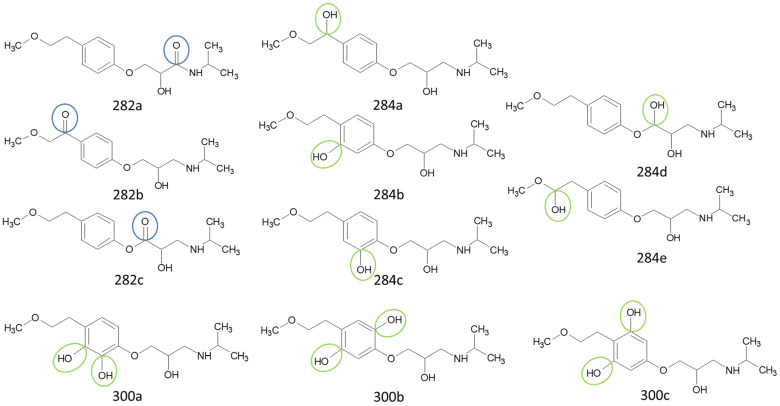
Structure proposals of degradation and transformation products from UV- irradiation and ozonation as used for QSAR analysis. Numbers correspond to rounded *m*/*z* values, index letters refer to isomers.

Prediction of toxicological and ecotoxicological parameters by QSAR is based on structural similarity. Hence, goodness of prediction heavily depends on the number of similar entries and their consistency. A recent study critically assessed in silico ecotoxicity prediction by comparison with in vitro testing [50]. In the current study, both QSAR software packages relied on the Simplified Molecular Input Line Entry System (SMILES™, https://www.daylight.com/smiles/index.html; accessed on 14 May 2021), which does not recognize tautomers due to the coding system and only takes stereochemistry into account in an extended version. Since hydroxyl addition or insertion may lead to regio-isomers and quinoid-like structures with metoprolol, isomers of the above-described degradation products were sketched and submitted to QSAR analysis. All structures presented in Table 2 and Table 3 were tested. Structures tested as isomers are shown in Figure 4. 

The results of ecotoxicity prediction using the OECD QSAR toolbox are listed in Table 4. According to the prediction, all products of photo-induced and hydroxyl radical induced degradation exhibited lower toxicity both acute and chronic against daphnid, fish, green algae, and *Pimephales promelas*, since the lethal concentrations (LC_50_) of all products were higher than that of metoprolol. Based on these results, no hazard to the aquatic environment should be expected when using UV radiation in the presence or absence of hydrogen peroxide to eliminate metoprolol. Among the ozonation products, only the product *m*/*z* = 206 was found to be of higher ecotoxicity to all organisms. As can be seen, isomers were predicted with different ecotoxicity. In case of structure family 300, hydroquinoid moieties within 300a and 300b provoked slightly lower toxic values than the meta-substituent arrangement within 300c. For the isomers of 282, the keto group was associated with less hazardous potential than the ester function, while for the amid isomer, no value was computed for branchiopoda, fish, and green algae. In general, larger and higher species seemed less prone to the adverse effects of metoprolol and its degradation or transformation products.

To compare the variation of prediction, Table 5 lists the corresponding values computed using VEGA. A total of 10 models were used within the OECD QSAR toolbox. Models based on the ECOSAR database employed structure specific training sets, whereas the model by Veith et al. and Pavan et al. relied on training sets and validation sets. As of today, the available data basis is limited. Exemplarily, the implemented data sets for acute toxicity prediction ranged from 22 to 83, for chronic toxicity from 3 to 13 points. The models by Veit et al. and Pavan et al. contained between 58 and 144 datasets. The goodness-of-fit could be determined by using training sets. The coefficients of determination were between 0.78 and 0.92. Using VEGA, some products, such as *m*/*z* = 252, were classified more toxic. The products with *m*/*z* = 284 and the one with *m*/*z* = 134 were predicted less toxic for most species, while no uniform trend could be identified for the other products. In contrast to the OECD QSAR toolbox prediction, the ozonation product was computed to be less ecotoxic. Furthermore, the evaluation of the isomers deviated as well. For the 300 family, the meta-isomer was considered less ecotoxic than the hydroquinoid isomers. The same applied for the 282 series. The amid isomer 282a could be predicted and was judged to be more hazardous than the keto isomer 282b. In VEGA, a total of 15 different models were used for the ecotoxicity prediction. They contained in general several hundred data on the ecotoxicity of various substances, from which the desired models are created. The larger amount of data allowed the prediction of the structure missing with the OECD toolbox. As a drawback, both toolboxes did not contain relevant experimental data on metoprolol nor on the transformation products as such. Similar compounds, however, were indicated. Yet, both toolboxes employed quality criteria mainly based on statistical parameters, which were retrieved in the QSAR model report format (QMRF). Which of the predictions would be more reliable could not be distinguished but would require experimental data, especially when different quality criteria were listed. Hence, the number of similar structures and consistent values used for model building and prediction is probably one of the most direct quantities for reliability evaluation, despite the deficiencies of the SMILES™ coding. In conclusion, both predictive tools yield qualitatively comparable results in general. Yet, uncommon and rare structures or novel compound classes lead to no or contradictory results. Nevertheless, a preliminary estimation of potential hazards from compounds putatively emerging during AOP induced elimination of micropollutants should be feasible. The results may be applied to evaluate the effectiveness of the AOP to remove conventionally hard-to-eliminate pharmaceuticals or other pollutants from wastewater effluents. To this purpose, the extension of ecotoxicological databases is necessary.

## 4. Conclusions

In this study, two different AOPs were examined to degrade metoprolol: UV-irradiation and ozonation. Hydrogen peroxide was found to accelerate the photo-induced degradation whereas pH had no significant effect. For ozonation, a saturated ozone solution led to faster metoprolol elimination than an ozone flow. Yet, the ozone-flow reactor arrangement might be optimized for similar performance. Overall, photo-irradiation proved the more efficient AOP. Although UV-irradiation may be technically more demanding, the use in WWTPs might be re-considered due to its efficiency and minimization of by-products. Capital and management cost, environmental impact, and safety risk would need to be balanced. Using HPLC-HRMS and MS/MS, secondary products could be elucidated. Due to excitation, hydroxyl radical and ozone reaction, degradation, and transformation products characteristic for the AOP and reaction mechanism were recognized. The secondary products were submitted to prediction of ecotoxicity using OECD QSAR toolbox and VEGA. In general, degradation products were predicted with lower ecotoxicity than metoprolol. Despite overall agreement of the two software tools, differences were found for isomers and ozonation products. While being valuable preliminary evaluation tools, databases for QSAR ecotoxicity need to be greatly extended in the future. The workflow to identify and monitor transformation products during AOP treatment by HPLC-HRMS, MS/MS and to assess their ecotoxicity by prediction software is suitable for other beta-blockers and pharmaceuticals.

## Figures and Tables

**Table 1:** Reaction rate constants and their half-lives of the degradation of metoprolol at pH 3, 6, and 9, and upon addition of H_2_O_2_.

pH	H_2_O_2_/mg L^−1^	*k*/min^−1^	*t* ½/min
3	-	0.20	3.44
6	-	0.18	3.76
9	-	0.18	3.94
6	10	0.55	1.26
6	30	1.14	0.61

**Table 2:** Photoinduced transformation products of metoprolol: Retention times, exact and accurate mass, and structure proposal.

Retention Time/min	[M+H] ^+^ _(exact)_	[M+H]^+^_(accurate)_	Proposed Structure	Reference
5.1	268.1907	268.1831	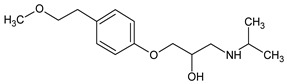	Metoprolol
2.9; 3.4; 4.1	284.1856	284.1796	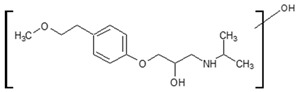	[30,31,32,33,34,35,36,37,38,39,40]
4.8; 5.0	282.1700	282.1612	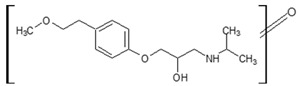	[30,31,32,33,34,36,37,38,39,40,41]
3.9	254.1387	254.1638	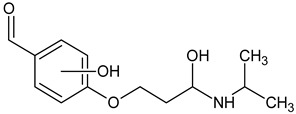	[30,31,32,33,34,35,37,38,40,41]
4.2	252.1594	252.1533	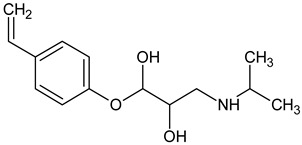	[30,31,33,34,37,38,40]
4.1	238.1438	238.1379	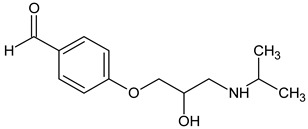	[30,31,33,34,35,36,37,38,39,40,42]
1.4	134.1176	134.1127	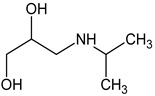	[30,31,32,34,35,36,37,38,39,40,41,43]

**Table 3:** Ozonation products of metoprolol: Retention times, exact and accurate mass, and structure proposal.

Retention Time/min	[M+H]^+^_(exact)_	[M+H]^+^_(accurate)_	Proposed Structure	Reference
5.9	268.1907	268.1872	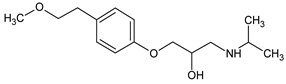	Metoprolol
5.3; 5.4	300.1805	300.1770	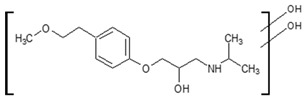	[30,31,34,39,40,47,48,49]
5.2; 5.8	282.1700	282.1703	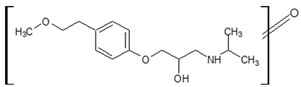	[30,31,32,33,34,36,37,38,39,40,41,45]
5.2; 5.3	274.1649	274.1615	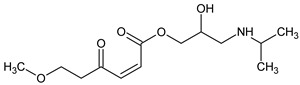	[47,48]
1.8	206.1023	206.0991	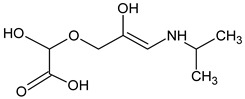	[34,48]
1.4	134.1176	134.1150	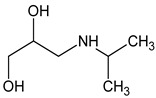	[30,31,32,34,35,36,37,38,39,40,41,43,48,49]

**Table 4:** QSAR analysis of metoprolol and with less (green) and more hazardous (red) degradation and transformation products from UV irradiation and ozonation using the OECD-QSAR toolbox.

	Daphnid (*Branchiopoda*)		Fish (*Actinopterygii*)		Green Algae		*Pimephales promelas* (Fathead Minnow)			
	48 h LC_50_/mg∙L^−1^	ChV /mg∙L^−1^	96 h LC_50_/mg∙L^−1^	ChV /mg∙L^−1^	96 h EC_50_ /mg∙L^−1^	ChV /mg∙L^−1^	M1—LC_50_/mg∙L^−1^	M2—LC_50_/mg∙L^−1^	M3—LC_50_/mg∙L^−1^	M4—LC_50_/mg∙L^−1^
Meto-prolol	9.44	0.75	82.1	5.33	8.36	2.71	144	208	437	111
134	170	10.1	2 × 10^3^	322	272	71.0	3.04 × 10^4^	1.25 × 10^4^	3.57 × 10^4^	3.99 × 10^3^
206	2.88	0.25	23.2	1.20	2.20	0.75	N.p.	46.8	90.9	28.4
238	15.5	1.17	142	10.8	15.2	4.73	228	420	928	205
252	17.8	1.34	164	12.8	17.7	5.50	161	497	1.11 × 10^3^	240
254	94.7	6.21	1 × 10^3^	119	124	34.8	N.p.	4.61 × 10^3^	1.18 × 10^4^	1.75 × 10^3^
274	107	6.96	1.13 × 10^3^	135	140	39.3	N.p.	5.26 × 10^3^	1.36 × 10^4^	1.99 × 10^3^
282 (a)	N.p.	N.p.	N.p.	N.p.	N.p.	N.p.	N.p.	1.44 × 10^3^	3.41 × 10^3^	627
282 (b)	65.4	4.46	666	69.8	79.1	22.8	N.p.	2.72 × 10^3^	6.7 × 10^3^	1.11 × 10^3^
282 (c)	38.5	2.74	375	34.3	42.6	12.7	N.p.	1.34 × 10^3^	3.15 × 10^3^	587
284 (a)	48.5	3.38	481	46.6	55.7	16.4	N.p.	1.81 × 10^3^	4.36 × 10^3^	772
284 (b)	19.6	1.47	180	13.9	19.4	6.01	N.p.	540	1.2 × 10^3^	262
284 (c)	27.7	2.03	262	22.1	29.1	8.84	N.p.	860	1.97 × 10^3^	397
284 (d)	48.5	3.38	481	46.6	55.7	16.4	N.p.	1.81 × 10^3^	4.36 × 10^3^	772
284 (e)	48.5	3.38	481	46.6	55.7	16.4	N.p.	1.81 × 10^3^	4.36 × 10^3^	772
300 (a)	57.3	3.96	573	57.2	67	19.6	N.p.	2.23 × 10^3^	5.40 × 10^3^	931
300 (b)	57.3	3.96	573	57.2	67	19.6	N.p.	2.23 × 10^3^	5.40 × 10^3^	931
300 (c)	40.4	2.87	393	35.9	44.6	13.3	N.p.	1.4 × 10^3^	3.29 × 10^3^	614

N.p. = Not predicted; other abbreviations given in the Experimental Section.

**Table 5:** QSAR analysis of metoprolol and its with less (green) and more hazardous (red) degradation and transformation products from UV irradiation and ozonation products using VEGA QSAR.

	Fish					Fathead Minnow		Daphnia Magna					Algae		
	Acute (LC_50_)/mg∙L^−1^				Chronic (NO EC)/mg∙L^−1^	LC_50_ 96 h/mg∙L^−1^	LC_50_/mg∙L^−1^	LC_50_ 48 h /mg∙L^−1^		Acute (EC_50_)/mg∙L^−1^		Chronic (NOEC)/mg∙L^−1^	Acute (EC_50_)/mg∙L^−1^		Chronic (NOEC)/mg∙L^−1^
	KNN/Read-Across 1.0.0	NIC 1.0.0	IRFMN 1.0.0	IRFMN/Combase 1.0.0	IRFMN 1.0.0	EPA 1.0.7	KNN/IRFMN 1.1.0	EPA 1.0.7	DEMETRA 1.0.4	IRFMN 1.0.0	IRFMN/Combase 1.0.0	IRFM 1.0.0	IRFMN 1.0.0	ProtoQSAR/Combase 1.0.0	IRFMN 1.0.0
**Metoprolol**	13.8	1.1	5.0	2.3	0.18	73.9	3.8	2.3	2.22	170	5.7 × 10^−3^	7.96	4.6	57.70	1.03
134	4019.8	512.0	15.6	268.5	3.40	1650.8	4091.4	1434.4	25.63	154	2.5	7.11	53.3	3.15	15.42
206	3056.1	93.7	17.4	64.1	0.65	694.1	N.p.	6727.0	393.32	102	1.11	10.4	21.2	26.84	18.96
238	13.0	3.4	4.7	3.2	0.25	102.6	13.5	4.7	7.51	44	1.4 × 10^−2^	7.72	5.3	0.85	2.17
252	16.7	3.6	5.4	1.7	0.15	40.7	12.7	50.6	6.32	4	3.3 × 10^−3^	7.73	4.1	0.74	1.52
254	28.0	5.2	4.5	2.5	0.29	14.6	27.0	64.1	47.49	450	0.18	7.39	9.9	0.08	1.09
274	1.76	19.9	3.31	3.0	0.31	222.1	0.6	9.0	6.5	751	0.36	9.18	8.0	101.64	1.78
282 (a)	14.0	1.1	8.7	1.8	0.19	63.8	4.1	1.9	6.20	217	1.9 × 10^−2^	10.09	4.9	63.79	1.16
282 (b)	21.7	1.1	5.2	2.8	0.18	88.6	4.5	2.2	7.43	285	1.7 × 10^−2^	11.16	4.4	0.69	0.98
282 (c)	29.6	1.1	7.9	3.0	0.20	47.1	13.0	1.9	1.41	188	4.9 × 10^−3^	8.77	4.7	0.67	1.23
284 (a)	35.8	1.2	6.4	4.4	0.19	212.7	4.3	45.0	7.40	335	1.3 × 10^−2^	10.96	4.2	0.69	1.50
284 (b)	36.7	1.2	5.8	7.6	0.19	81.4	4.5	40.9	22.11	276	2.0 × 10^−3^	8.33	5.8	62.70	1.13
284 (c)	16.4	1.2	6.6	6.6	0.19	91.4	4.4	35.4	22.63	237	6.5 × 10^−3^	9.49	5.4	10.36	1.04
284 (d)	35.7	1.2	7.2	4.6	0.22	89.9	4.3	38.5	6.49	202	3.7 × 10^−3^	11.37	5.3	0.67	1.13
284 (e)	35.8	1.2	5.6	8.2	0.19	132.6	4.3	47.3	5.78	7	1.7 × 10^−2^	9.79	4.9	53.5	1.13
300 (a)	17.1	36.6	4.8	5.7	0.27	86.3	23.7	647.0	82.16	322	2.4 × 10^−3^	9.82	7.9	10.13	1.29
300 (b)	17.1	36.6	5.7	4.1	0.23	91.3	23.7	722.3	81.5	394	2.4 × 10^−3^	9.41	8.6	12.04	1.06
300 (c)	36.9	36.6	5.5	4.2	0.18	82.2	4.9	793.9	75.5	314	8.0 × 10^−4^	7.6	13.5	0.76	1.48

N.p. = Not predicted; other abbreviations given in the Experimental Section.

## Data Availability

The data presented in this study are available on request from the corresponding author. The data are not publicly available due to restrictions by Sciebo. The link will be provided upon request.
